# Multi-modal transformer architecture for medical image analysis and automated report generation

**DOI:** 10.1038/s41598-024-69981-5

**Published:** 2024-08-20

**Authors:** Santhosh Raminedi, S. Shridevi, Daehan Won

**Affiliations:** 1 School of Computer Science and Engineering, Vellore Institute of Technology, Chennai, India; 2 Centre for Advanced Data Science, Vellore Institute of Technology, Chennai, India; 3 Department of Systems Science and Industrial Engineering, The State University of New York (SUNY), Binghamton University, Binghamton, USA

**Keywords:** Vision transformer, Generative pre-trained transformer, Retrieval augmentation, Diseases, Respiratory tract diseases, Respiratory distress syndrome

## Abstract

Medical practitioners examine medical images, such as X-rays, write reports based on the findings, and provide conclusive statements. Manual interpretation of the results and report generation by examiners are time-consuming processes that lead to potential delays in diagnosis. We propose an automated report generation model for medical images leveraging an encoder–decoder architecture. Our model utilizes transformer architectures, including Vision Transformer (ViT) and its variants like Data Efficient Image Transformer (DEiT) and BERT pre-training image transformer (BEiT), as an encoder. These transformers are adapted for processing to extract and gain visual information from medical images. Reports are transformed into text embeddings, and the Generative Pre-trained Transformer (GPT2) model is used as a decoder to generate medical reports. Our model utilizes a cross-attention mechanism between the vision transformer and GPT2, which enables it to create detailed and coherent medical reports based on the visual information extracted by the encoder. In our model, we have extended the report generation with general knowledge, which is independent of the inputs and provides a comprehensive report in a broad sense. We conduct our experiments on the Indiana University X-ray dataset to demonstrate the effectiveness of our models. Generated medical reports from the model are evaluated using word overlap metrics such as Bleu scores, Rouge-L, retrieval augmentation answer correctness, and similarity metrics such as skip thought cs, greedy matching, vector extrema, and RAG answer similarity. Results show that our model is performing better than the recurrent models in terms of report generation, answer similarity, and word overlap metrics. By automating the report generation process and incorporating advanced transformer architectures and general knowledge, our approach has the potential to significantly improve the efficiency and accuracy of medical image analysis and report generation.

## Introduction

In modern medicine, radiology plays a vital role in diagnosis and treatment planning for a disease. Radiological Imaging techniques such as X-ray and magnetic resonance imaging (MRI) provide valuable insight into the human body and allow clinical practitioners to visualize and diagnose the medical problem. Radiological image interpretation can be complex and very time-consuming and also requires experts^1^. In recent advancements in AI technology, significant research is conducting to streamline the process of automating medical report generation from radiology images. Many Deep learning frameworks have been developed recently to automate the report generation of medical images. They aim to produce accurate insightful comprehensive reports to reduce the manual processing time. Understanding radiology reports and providing assistance is time-consuming and difficult for radiologists. The rapid evolution of deep learning revolutionized radiology analysis in extracting features and patterns from complex X-ray datasets^2^.

Recognizing the need for more efficient and accurate reporting methods, researchers have turned to advanced deep-learning architectures to address the limitations of manual reporting. Building upon the convolution-recurrent architectures (CNN-RNN) commonly used in image captioning research, recent studies have explored integrating visual attention mechanisms and transformer-based models for automatic report generation. Transformers, known for their parallelizability and superior performance in natural language processing tasks, offer a promising avenue for automating report writing in the medical domain. The evolution of Natural language processing techniques has seen a paradigm shift towards transformer-based models, which excel in learning contextual relationships and generating coherent text. By fine-tuning pre-trained transformer models such as GPT-2 on large corpora of medical imaging data, researchers aim to leverage the generative capabilities of these models for medical report generation. Conditioning the transformer on visual features and semantic tag embeddings allows for seamless integration of image information into the text generation process, enabling more accurate and contextually relevant reports^1,3^.

In this paper, we propose a novel multi-modal-based transformer architecture with the Vision transformer as an encoder and Generative pre-trained transformer 2 as a decoder, which we call ViGPT2. Vision transformer as a feature extractor has the advantage of capturing the global context of the images effectively^4^. In CNN-based feature extractor processes images hierarchically, extracting the local features only. Whereas ViT processes the image into small patches, and by projection, it will transform the patches into vector embedding allowing the dependencies more efficiently. It has multi-head self-mechanism which allows it to focus on the relevant patches of the medical image and pass to the multi-head self-attention and MLP blocks. Layer Normalization (LN) is applied before every block and residual connections after every encoder block. Along with the vision transformer we have worked on its variants like BERT pre-training image Transformer (BEiT) and Data Efficient Image Transformer (DEiT). BERT pre-training image Transformer with GPT2, we call BEiTGPT2. Similar to the Vision transformer Beit converts images into patches. BERT pre-training image transformer reduces the computational complexity of the ViT, by down striding mechanism. In this mechanism spatial resolution of the input feature is reduced and self-attention and the resolution afterward which increases the number of attention operations decreases and improves the efficiency of the feature exactor. Data efficient image transformer, we call DEiTGPT2 enhances the data efficiency of the ViT. DEiT employs data augmentation during pre-training which increases the diversity of the data and improves the model’s robustness to variations in the medical images^5^. GPT2 as a decoder converts the textual medical reports into tokens and the self-attention mechanism enables the model to create the dependencies between the tokens. The encoder output using cross attention mechanism model maps the features of the medical images to the tokens^6^. After obtaining enriched representations from both self-attention and cross-attention mechanisms, our model is conditioned on image features and text embeddings to generate full-text reports.

The significant contributions of our work are mentioned below:

1. We proposed a novel multi-modal transformer-based architecture with Vision transformer and Generative Pre-trained Transformer 2, which outperformed the existing works.
2. ViT, BEiT, and DEiT are used as a feature extractor for medical images.
3. We have used Cross cross-attention mechanism between the structural information of the medical reports and image features.
4. Our models are evaluated with word overlap metrics and semantic answer similarity (SAS) metrics which show the quantitative performance.
5. Reports are enhanced with general knowledge through the use of Chroma vector store and Lang chain by Retrieval augmentation.

## Discussion

The literature on automated report generation from medical images reveals a dynamic landscape characterized by diverse methodologies and evolving technologies. Table 1 shows the contributions of various researchers in the medical report generation domain. Yuan et al.^7^ proposed a framework, MvH, that uses encoder–decoder architecture. Yuan et al. use a multi-view CNN encoder and conceptually enriched hierarchical LSTM decoder. In this framework, they proposed three task schemes: first, pre-train the images with an encoder, then extract crucial information from the medical reports^7^. Finally, put up images and text information in the model and generate the reports. Yang et al., in their study, worked on a multi-modal approach for radiology report generation. Yang et al.^8^ worked on the IU-X-ray and MIMIC-CXR datasets, and they worked on an approach in which observations in reports are highly related to the features in the images. Their framework consists of two modules: the knowledge base module, which extracts the textual embeddings from the reports, and the multi-modal alignment module, which applies the features of the X-ray and disease labels^8^.

**Table 1:** Researchers contribution.

Dataset	Reference	Bleu1	Bleu2	Bleu3	Bleu4	CIDEr	ROUGE-L
IU-Xray	Yuan et al.^7^	0.529	0.315	0.255	0.343	–	0.453
	Yang et al.^8^	0.497	0.319	0.230	0.174	0.407	0.399
	Xue et al.^9^	0.464	0.358	0.270	0.195	–	0.366
	Chen et al.^10^	0.470	0.304	0.219	0.165		0.371
	Changchang et al.^11^	0.436	0.278	0.197	0.150	0.381	0.341
	Shuxin et al.^12^	0.496	0.327	0.238	0.178	0.382	0.381
	Yaowei et al.^13^	0.530	0.365	0.263	0.200	0.501	0.405
	Mohsan et al.^14^	0.532	0.344	0.233	0.158	0.50	0.387
	Christy et al.^15^	0.482	0.234	0.143	0.096	0.280	0.339
	Srinivasan et al.^7^	0.464	0.301	0.212	0.158	–	–
	Fenglin et al.^8^	0.360	0.224	0.149	0.106	–	0.284
MIMIC-CXR	Yang et al.^2^	0.386	0.237	0.157	0.111	0.111	0.274
	Chen et al.^5^	0.353	0.218	0.145	0.103	-	0.277
	Manuela et al.^6^	–	–	–	–	–	0.373
	Shuxin et al.^12^	0.363	0.228	0.156	0.115	0.203	0.284
	Yaowei et al.^13^	0.363	0.229	0.158	0.107	0.246	0.289
	Fenglin et al.^8^	0.483	0.315	0.224	0.168	–	0.351
Che-X-Net	Alfarghaly et al.^16^	0.387	0.245	0.166	0.111	0.257	0.289

Xue et al.^9^ proposed a model for automatic radiology report generation using a multimodal recurrent network with an attention mechanism. They have integrated CNN with LSTM in a recurrent manner. The Xue et al. model is capable of not only generating high-level conclusive impressions but also generating detailed descriptive findings sentence by sentence to support the conclusion. Furthermore, a multimodal model combines image encoding and generated sentences to construct an attentional input that controls the generation of the next sentence and ensures consistency between the generated sentences. Shin et al.^17^ proposed a model to annotate the chest X-ray images. In their model, they worked on a CNN/RNN-based architecture to annotate the images. They adopted many regularization techniques to work on the normal vs. disease bias. RNN is used to learn the annotated sequence of input image embeddings. Shin et al. tested Long Short-Term Memory (LSTM) and Gated Recurrent Unit (GRU)-based implementations of RNNs^17^.

Chen et al.^10^ generate reports using a memory-driven transformer. In their proposed method, a relational memory is used to capture the essential information of the reports for generation, enhancing the decoder’s performance through memory-driven conditional layer normalization. The model structure comprises a visual extractor using a state-of-the-art model (CNN), a standard transformer encoder, and a decoder with an integrated memory module^10^. Manuela et al.^18^ proposed an approach for radiology report generation that is a two-step method that primarily detects abnormalities in chest X-ray (CXR) images. This initial step addresses a multi-class problem by localizing identified abnormalities with bounding boxes and associated probability scores and detecting various lung lesions such as nodules, masses, and pneumothorax in X-rays^18^. Abnormality lesions and corresponding probabilities are transformed into textual embeddings, and then a large language model is fine-tuned for the findings and to make a comprehensive radiology report.

Yin et al.^11^ proposed a novel framework using a hierarchical recurrent neural network (HRNN) with a soft attention mechanism for report generation. They have used the image captioning approach with the topic matching approach to produce a detailed description of the trained image. The abnormalities and captions are transformed into sentence semantic embeddings and fed into HRNN^11^. Alfarghaly et al.^6^ introduce a novel architecture, CDGPT2 (Chest X-ray dataset finely tuned with GPT2), to automate the generation of radiology reports using chest X-ray images. The Chexnet model is used as a visual extractor that acts as an encoder and extracts tags from the images. Semantic feature extraction analyzes the weighted semantic features of the images. Based on the visual and semantic embeddings, the GPT2 model is trained to generate reports.

Shuxin et al.^19^ model introduces a framework that integrates general knowledge (input-independent) and specific knowledge (input-dependent) for report generation for the X-ray dataset. Shuxin et al. framework works on the concept of multi-head attention with a knowledge-enhancing approach and also integrates the visual features of the images with a knowledge base to outperform the other models^19^. Yaowei et al.^20^ proposed a Unify, Align, and Refine (UAR) approach to improve and learn multi-level cross-modal alignments. Three modules were introduced in this framework: the Latent Space Unifier (LSU), the Cross-Modal Representation Aligner (CRA), and the Text-to-Image Refiner (TIR).

Mohsan et al.^21^ proposed the TrMRG model, which is an encoder-decoder integrated with transformer architecture. The encoder in this model consists of a stack of identical layers to process and break the images, and information is extracted using self-attention heads to apply positional encoding to the information^2^. The decoder receives encoded features as queries and keys, predicts word probabilities, and passes them on through a linear layer and SoftMax, producing hidden states or latent space to generate reports for the chest X-ray dataset. Kisilev et al.^15^ introduced a framework for automatic breast radiology report generation. They have used structural learning by extracting and training with lesions. The proposed model consists of three modules: lesion detection, an image feature extractor, and a semantic layer that generates the probability of a lexicon for a particular image. Standard lexicons and feature extractors help in understanding the CAD system’s diagnosis and detection^15^.

Christy et al.^22^ introduce a “knowledge-driven encode, retrieve, paraphrase (KERP)” framework. KERP dissects the medical reports into abnormalities; an encoder is used to extract the visual information and abnormalities in the images; and then Graph Transformer transforms the text embeddings into graph-structured data. Srinivasan et al.^23^ propose a two-stage divide-and-conquer approach in their work. Initially, abnormal patients’ reports were separated, and tags were extracted from their reports. Unlike other frameworks, they have introduced a unique transformer architecture that consists of two encoders for tag embeddings and image features, and two decoders are stacked to learn and improve the reports. Fenglin et al.^24^ models work on posterior and prior knowledge of the dataset in report generation. The modules that were proposed in the framework are “Posterior Knowledge Explorer (PoKE), Prior Knowledge Explorer (PrKE), and Multi-Domain Knowledge Distiller (MKD)”^24^. Their work analyzes the textual bias with prior knowledge and generates comprehensive reports.

## Methodology

**Figure 1:**
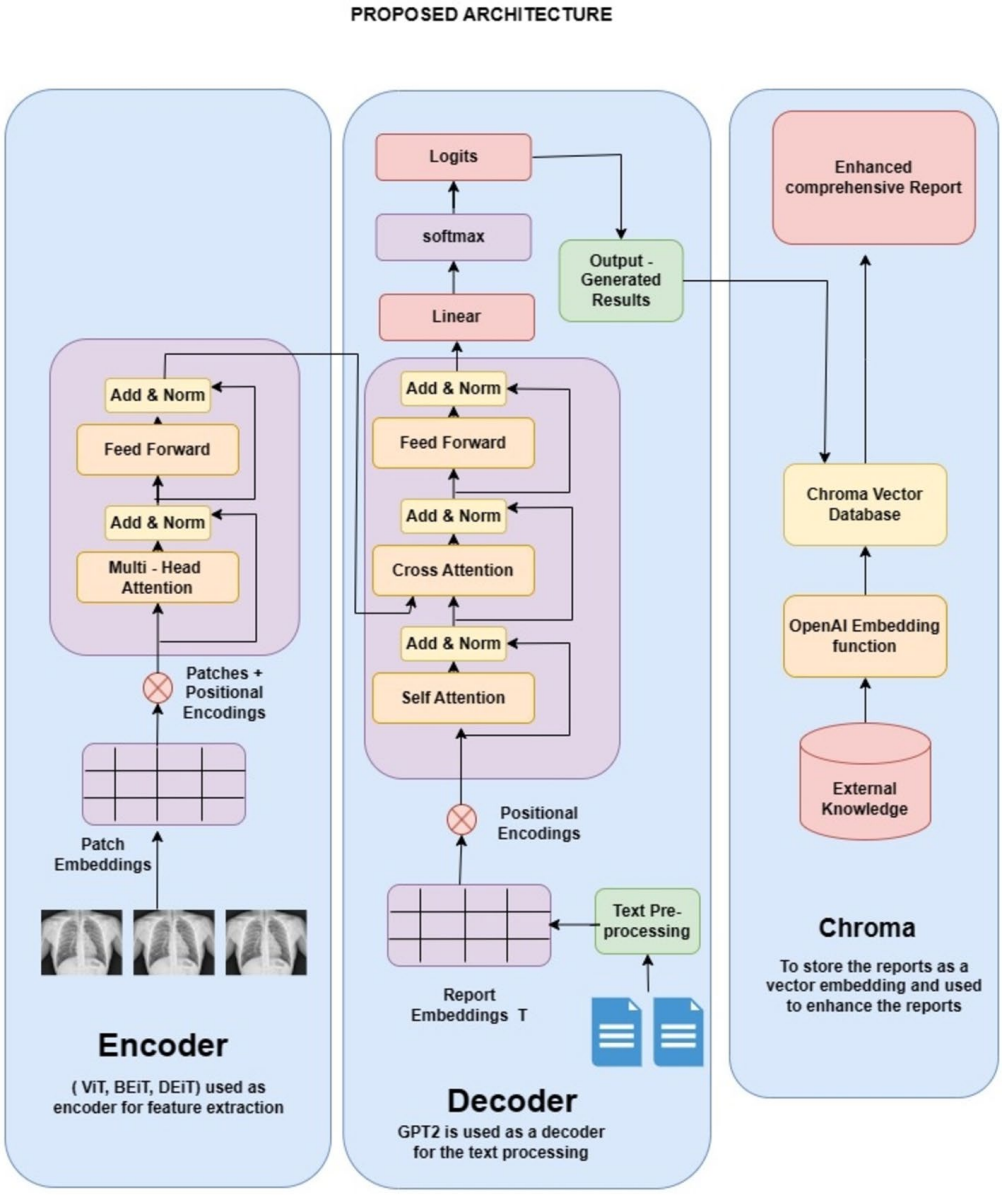
Proposed architecture.

The proposed architecture consists of three major components (a) a Transformer Encoder to predict the abnormalities in the medical image and process it into trainable visual features, (b) GPT2 as a decoder to train on the textual embeddings of the report with the visual features to generate comprehensive medical findings and (c) Chroma vector store and lang chain module for retrieval augmentation of the findings generated by decoder. The Fig. 1 describes the proposed model for report generation.

The novelty of our architecture lies in terms of the feature extraction of the Vision transformer and the retrieval augmentation for enhancing the reports. Most of the architectures for feature extraction rely on CNN-based convolutional filters to extract features, whereas ViT utilizes a self-attention mechanism. This mechanism analyses relationships between different parts of the image, allowing it to capture long-range dependencies and global context more effectively^25^. ViT divides the input image into smaller patches. These patches are then fed through a linear projection layer to embed them into a lower-dimensional vector space. This step allows the model to process information from each patch independently before attending to their relationships. These layers employ the self-attention mechanism to progressively extract features and build a richer representation of the image^4^. Retrieval augmentation of the findings has several advantages by incorporating similar reports retrieved from Chroma, our proposed work has access to a factual knowledge base, reducing the risk of generating entirely fabricated information and also it can draw insights from multiple reports, the generated report likely to be more comprehensive and trustworthy, boosting its overall credibility^16^.

### Dataset

We are using the Open-I collection of the Indiana University X-ray dataset from the Indiana University hospital network as a base dataset for medical Imaging analysis and report generation work^26^. This dataset contains 7470 X-ray images originally in the Dicom standard form which is a representation of the digital medical images and 3851 patients reports. Every image in the dataset consists of two views frontal and lateral view. The number of X-ray images per report varies from 1 to 5. The Table 2 describes the number of images associated with the reports.

**Table 2:** Images associated with reports.

Number of views	Number of reports	Number of images
1	446	446
2	3208	6416
3	181	543
4	15	60
5	1	5

Our training data resides in a dataset containing three key elements: indication, impression, and findings (Fig. 4). To prepare this data for model training, we meticulously pre-processed each column. This involved a series of steps designed to clean, normalize, and potentially transform the data to ensure its suitability for the model. Following this pre-processing, we leveraged the processed data to create a new, highly informative attribute: a summary of findings. This summary attribute condenses the key insights gleaned from the original data points, providing a more efficient and cohesive representation for the model to utilize during the training process.

### Data preprocessing

**Figure 2:**
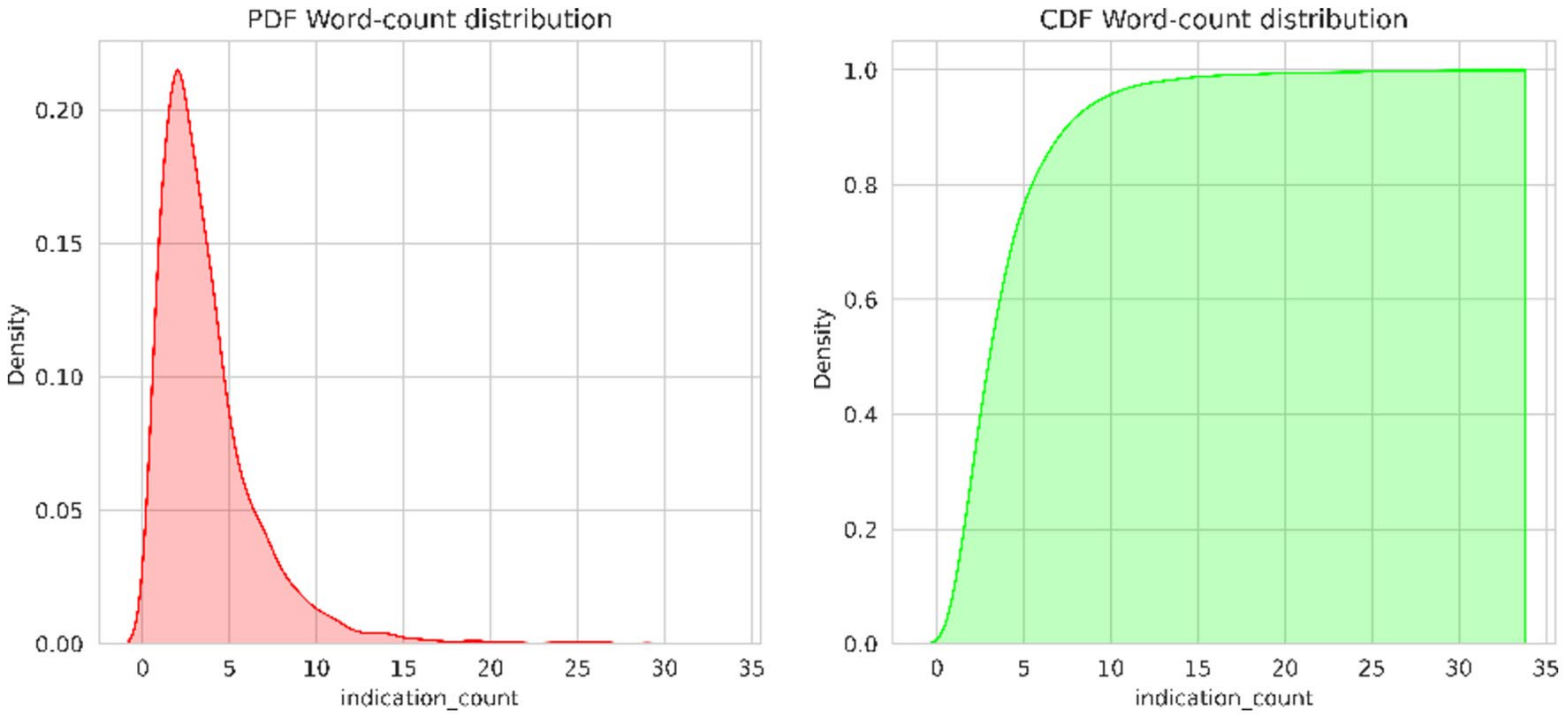
Indication probability density function (PDF) and cumulative density function (CDF).

**Figure 3:**
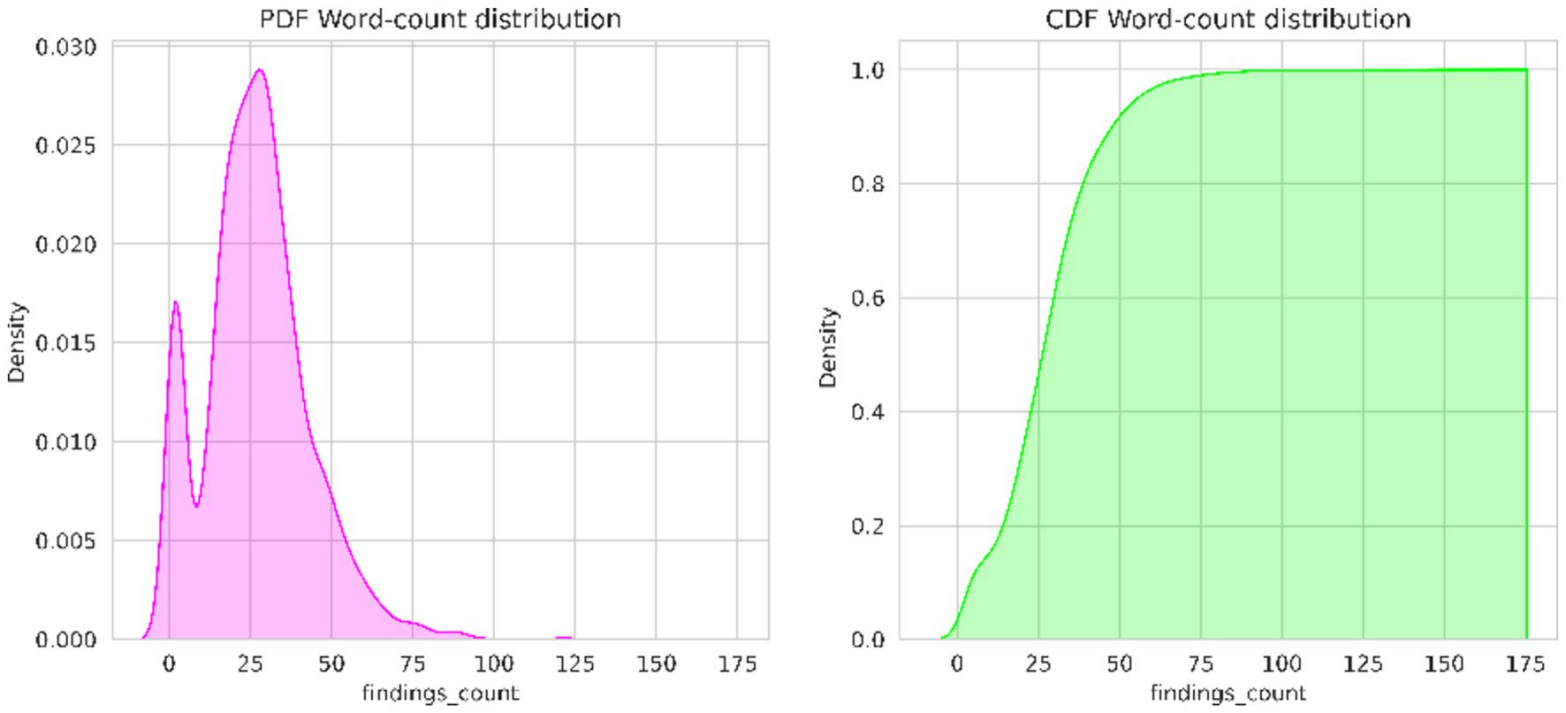
Findings probability density function (PDF) and cumulative density function (CDF).

**Figure 4:**
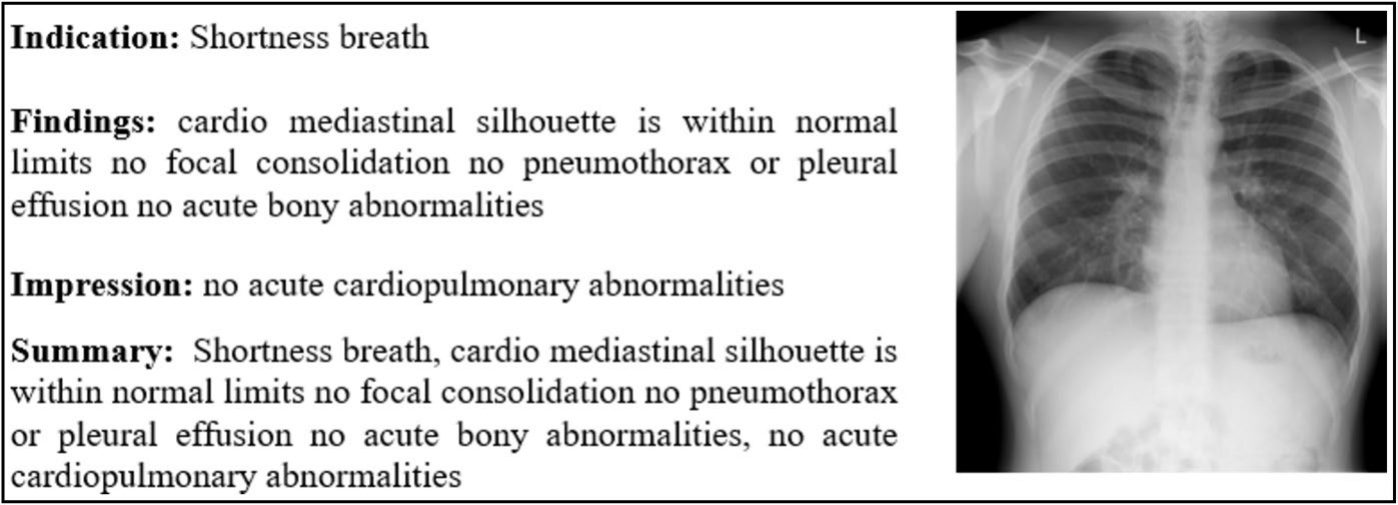
Sample data (Image and summary are used for model training).

In this step, the dataset undergoes to preprocessing to handle missing values and extract the relevant abnormalities feature. It is observed that comparison, indication, and columns consist of Nan values. Values like “No comparison”, “No indication”, “No findings” and “No impression” were added to those columns. The indication feature provides valuable insights for the medical examination. The indication column minimum and maximum word count are 2 and 32 respectively. From the probability density function and cumulative density function of the Fig. 2. It is observed that 50% of the indications consist of 4–5 words, and 99% of the indications contain fewer than 10 words. From the word cloud analysis highlighted key terms are chest, pain, shortness, dyspnea, etc. Similarly, the findings column minimum and maximum word count are 1 and 123 respectively, medium sentence length is 5. From the PDF and CDF of Fig. 3, it is observed that 50% of the data have less than 25 words, 99% data have less than 50 words, and only 1% data contains more than 50 words per sentence. From the word cloud analysis of the findings, the highlighted key terms are pleural effusion, pneumothorax, heart size, cardio mediastinal silhouette, mediastinal contour, mediastinum, etc. (Fig. 4).

### Encoder for feature extractor

Encoder Transformer takes input image X $\in$ ${\mathbb{R}}$^*H X W X C*^ then the image is reshaped into a set of 2D flattened patches X_p_ $\in$ ${\mathbb{R}}^{N\times \left({P}^{2}\cdot C\right)}$ where (H, W) is the resolution of the original image, C is the number of channels, (P, P) is the resolution of each image patch, and $N=\frac{HW}{{P}^{2}}$ is the resulting number of patches. Each patch is converted into the low dimensional vector by projecting the patch into the vector embeddings dimension as Eq. (1). Generally, transformers don’t possess information about the spatial relationship between the arrangements of tokens, positional embeddings are crucial for preserving spatial information of the image^25^. Therefore, positional embeddings are added to Patch embeddings. Each patch embedding augmented with positional embeddings is concatenated along the patch dimension to form a sequence of token embeddings. Token embeddings are fed into the encoder block for image processing. These blocks contain a series of layers multi-head self-attention, multi-layer perceptron, and Layer Normalization as Eqs. (2)–(4). The output of the encoder is passed to the decoder block for relational mapping of image features and text features.

1 $${{\varvec{z}}}_{{\varvec{o}}}= \left[{x}_{class}; {x}_{p}^{1}{\varvec{E}} ; {x}_{p}^{2}{\varvec{E}}; . . . ; {x}_{p}^{N}{\varvec{E}}\right] + {{\varvec{E}}}_{{\varvec{p}}{\varvec{o}}{\varvec{s}}} ,{\varvec{E}}\in {\mathbb{R}}^{\left({P}^{2}.C\right) x D} , {{\varvec{E}}}_{{\varvec{p}}{\varvec{o}}{\varvec{s}}} \in {\mathbb{R}}^{\left(N + 1\right) x D},$$

2 $${z}_{{\ell}}^{\prime} =\text{ MSA }\left(LN \left({z}_{{\ell}-1}\right)\right) + {z}_{{\ell}-1},{\ell}=1\dots L,$$

3 $${z}_{{\ell}} = MLP \left(LN \left({z}_{{\ell}}^{\prime}\right)\right) + {z}_{{\ell}}^{\prime},{\ell}=1\dots L,$$

4 $$y = LN\left({z}_{L}^{0}\right).$$

### Decoder for text generation

A GPT2 (Generative pre-trained Transformer 2) based architecture is used as a decoder in our proposed model. The input to the GPT2 decoder is the medical reports of the X-ray images. Reports are in XML format which is pre-processed into text format. The input text is tokenized into tokens by decoder tokenizer. Token embeddings are typically represented as X = [× 1, × 2,…, xn], where xi represents the embedding vector for the i-th token and positional encodings are added to token embeddings to capture the information about the sequence of tokens^27^.

#### Self-attention mechanism

In the decoder block, the self-attention mechanism enables to capture of the dependency and relationship between the sequence of the tokens. It allows to model to calculate the weight of the importance of different medical words in the medical reports^28^. Each word in the input sequence is associated with the three vectors: Query, Key, and value which are learned during the model training. Query = X W^Q^, Key = X W^K^, Value = X W^V^, where X is the input sequence, and W^Q^, W^K^, and W^V^ are learned weight matrices^27^. Attention score is calculated using the below Eq. (5), which determines the importance of each token concerning others. High attention indicates more relevance to the current step.

5 $$Attention=softmax\left(\frac{Q{K}^{T}}{\sqrt{{d}_{k}}}\right)V,$$

where Q, K, and V are the Query, Key, and Value matrices respectively, and *d*_k_ is the dimension of the Key vectors.

#### Cross-attention mechanism

In Cross Attention mechanism, the model tries to incorporate the visual information of the X-ray image. This involves extracting the output of the vision transformer (ViT) from the encoder and calculating the attention score^15^. In the attention Mechanism, the attention score is calculated from the self-attention score and encoder output^28^ as shown in Eq. (6). During cross-attention, each token in the report’s modality attends to relevant visual features which are represented by the Output of the encoder^29^. This allows the VIGPT model to integrate relevant visual information into the text generation process^11^.

6 $$cross\, Attention=X+softmax(\frac{{Q}_{decoder }{K}_{encoder}^{T}}{\sqrt{{d}_{k}}}){V}_{encoder.}$$

#### Position-wise feedforward neural network

In the decoder after the contextual representation of the image and text vectors, the decoder block applies a position-wise feed-forward network. In this Feed-forward network, there is a series of fully connected layers with an activation function GELU Eq. (7), and it is applied after each linear transformation^14^. This position-wise feed-forward network enables to capture and retrieval of the relation between the X-ray image abnormalities and specific keywords and redefines the vector representation^3^.

7 $$GELU\left(x\right)=x\cdot \varphi \left(x\right),$$

where $\varphi (x )$ is the standard cumulative distribution function (CDF) of the standard normal distribution, given by Eq. (8):

8 $$\varphi \left(x\right)= \frac{1}{2}\left(1+\text{erf}\left(\frac{x}{\sqrt{2}}\right)\right).$$

Here, erf(x) denotes the error function, which represents the integral of the Gaussian (normal) distribution.

In the decoder block, layer normalization, and residual connections are present. It helps to stabilize the model training and update the sequence weights. Layer normalization normalizes the inputs to each layer^30^. It helps to mitigate the issue of internal covariate shift and also improves the training stability. Residual connections allow gradients to flow more directly through the network. During training by providing shortcuts for gradient propagation^2^. This helps alleviate the vanishing gradient problem and enables more efficient training.

#### Output generation

Finally, the decoder generates a sequence of tokens from the given input images. The final decoder block produces a logit over on a trained medical report, which is a representation of the model’s predictions for the next token in the sequence. Logits are vectors which are some values. A SoftMax function is applied to the logits to convert those values into a probability distribution over a sequence of possible tokens^31^. This makes the model generate the next token in the sequence from the medical X-ray image. From the generated sequence of tokens, the model tries to make a sentence about the findings of the medical image^32^.

### Chroma vector store and lang chain

To generate a comprehensive medical report, chroma a vector database is used for storing the additional knowledge and medical reports as a vector. Lang chain is used for the retrieval augmentation of the generated finding from the ViTGPT2 model using Chroma Vector Store. Chroma optimizes storage for vector embeddings, allowing for efficient retrieval and it is cost-effective when compared to the FAISS and Pinecone. Chroma is an Open-source vector store and acts as a storage retrieval system^16^. The prompt template used for the retrieval Augmentation guides the LLM for the specific aspects of the findings. The prompt template consists of an indication, impression, and summary of the findings. Indication: Based on the findings, what are the key indicators or observations? Impression: What is the overall impression or feeling conveyed by the findings? Summary of findings: Provide a concise and informative summary of the most important findings.

In this module, our approach combines Lang Chain, a data pipeline tool, with Chroma, a vector store, and prompt templates to unlock deeper insights from ViTGPT2-generated findings. ViTGPT2 provides an initial analysis, stored in Chroma. Prompt templates then guide a large language model to analyze specific aspects of the findings, like key indications or a concise summary. By retrieving similar findings from Chroma and applying these prompts, the system generates more nuanced insights, improving efficiency and understanding.

## Results

Vision Transformer is a novel transformer for image analysis that processes medical images into self-attention mechanisms^12^. The input image is divided into fixed-size regions called patches. This allows the model to process information from smaller local areas of the image independently. Each patch is then passed through a linear projection layer, transforming it into a lower-dimensional vector representation. This embedding process captures essential information within each patch. The embedded patches are then fed into a series of Transformer encoder layers. These layers employ a self-attention mechanism, which allows the model to analyze relationships between different patches, capturing long-range dependencies and global context within the image.

BEiT (Bidirectional Encoder Representations from Image Transformers) is exactly the approach that applies BERT-style pre-training to image transformers. Similar to how BERT masks words in a sentence, BEiT masks image patches within an image^4^. The model then predicts the masked content based on the surrounding unmasked patches. This process encourages the model to learn relationships between different parts of the image and develop a strong understanding of the image context. Unlike the standard ViT which uses a unidirectional encoder, BEiT employs a bidirectional Transformer encoder. This allows the model to process information in both directions, further enhancing its ability to capture relationships and context within the image.

**Figure 5:**
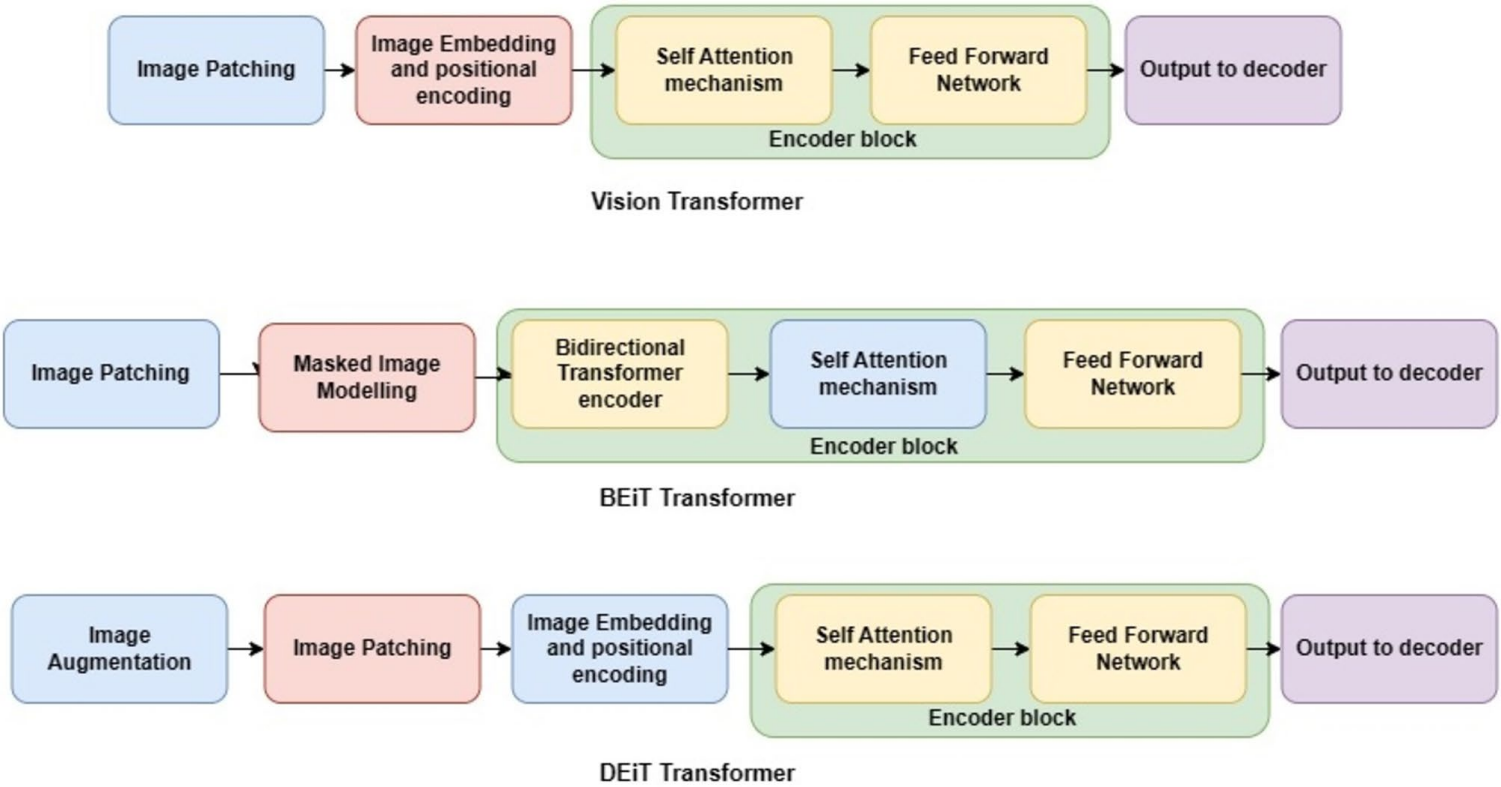
Encoder architectures of (ViT, BEiT, DEiT).

DEIT (Data Efficient Image Transformer) is a vision transformer architecture specifically designed for training with limited labeled image data. Similar to the standard ViT architecture, DEIT utilizes a Transformer encoder as its backbone for feature extraction^2,5^. The image is divided into patches, which are then embedded into a lower-dimensional vector space. DEIT employs a specific variant of the Transformer encoder with slightly modified feed-forward layers for improved efficiency^13^. DEIT models can be computationally expensive to train compared to some CNN architectures. However, their data efficiency allows them to achieve good performance with less training data, potentially reducing overall training costs. Figure 5 shows the architecture diagram of these three encoders.

Encoder architectures are used as feature exactors for the medical images Fig. 5 shows the encoder architectures used in our model. It consists of stacked encoder layers of 12 blocks. Medical images were resized into 224 × 224 and transformed the image into non-overlapped patches of 16 × 16 size for model training^14^. The dimensionality of the token embeddings is set to 768, to determine the feature representation, and also positional embeddings are typically set to 768 dimensions. The dimensionality of the feed-forward network is set to 3072, which determines the model to capture information and feature mapping. A batch of size 32 with Adam optimizer and learning rate of 1e-4 and drop out a layer of 0.1.

GPT2 is used as a decoder which has the same configuration of expansive vocabulary size of 50,257 enabling the decoder model to comprehend and generate a diverse range of tokens of the medical reports. The embedding dimension set at 768 enables the representation of input tokens extracting the semantic relationship between text tokens and image embeddings. With 12 attention heads, the model exhibits a remarkable capacity for parallelized processing.

**Figure 6:**
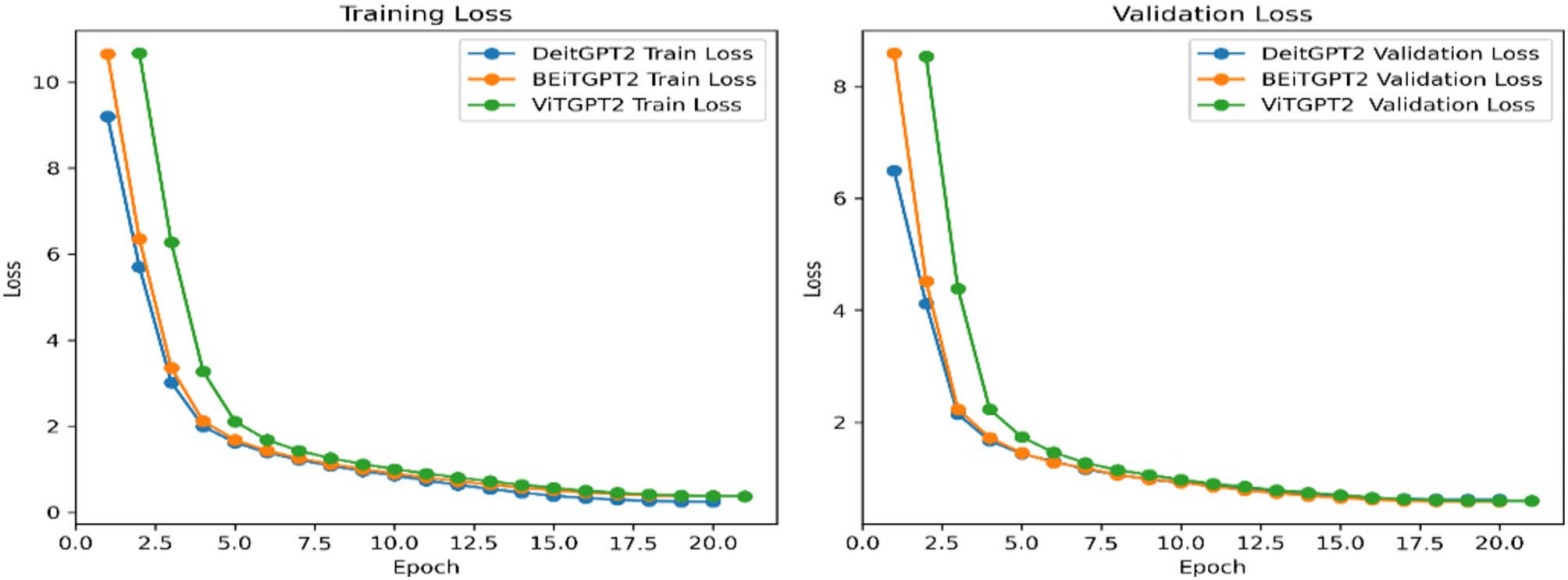
Training and validation loss.

**Figure 7:**
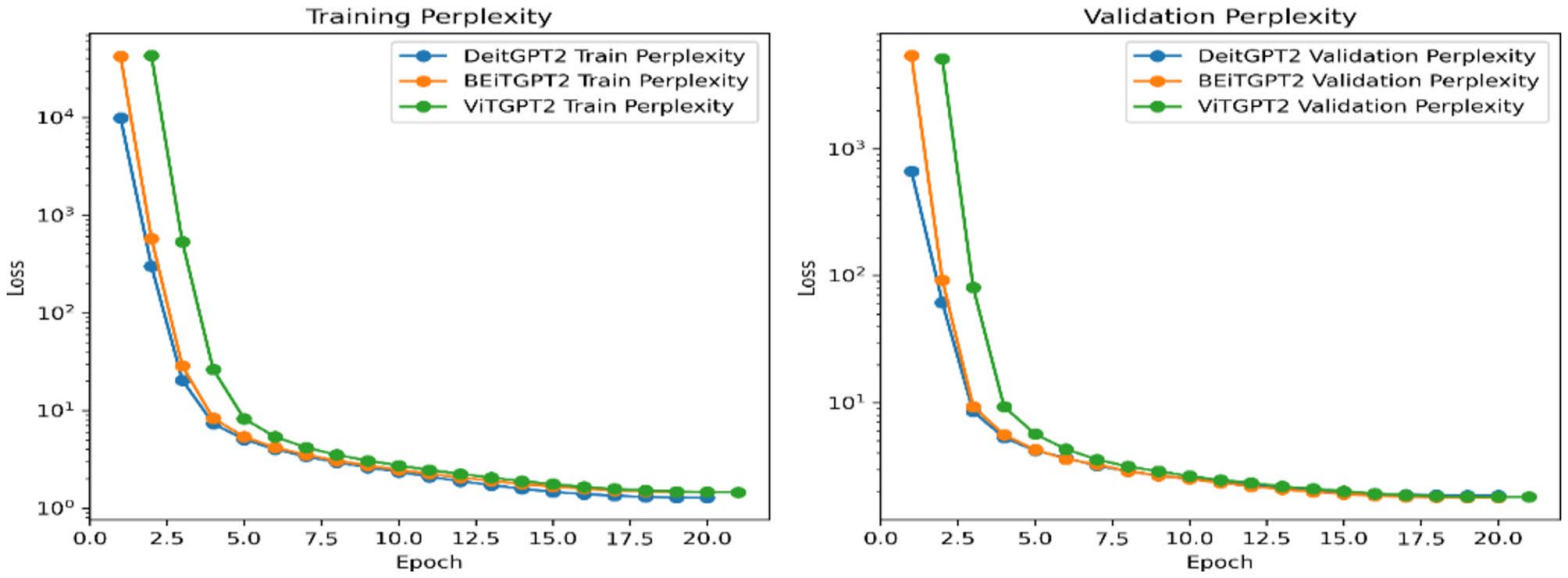
Training and validation perplexity.

The sequence length, capped at 1024, provides the flexibility to handle different medical terms in the report which enables the model to adapt to a wide range of input text. The architecture’s depth, a crucial determinant of its capacity to capture complex patterns, is precisely defined with 12 transformer blocks. The inclusion of attention and residual dropouts at a rate of 0.1 introduces a regularization mechanism. The utilization of an MLP ratio of 4 reflects a nuanced understanding of the trade-off. Between model expressiveness and computational efficiency. Adding a 0.1 dropout to the MLP layer further refines the model’s adaptability, enhancing its capacity to handle varying degrees of complexity in input data. Figure 6 shows the training and validation loss of proposed architectures and Fig. 7 shows the training and validation perplexity.

### Quantitative analysis

**Figure 8:**
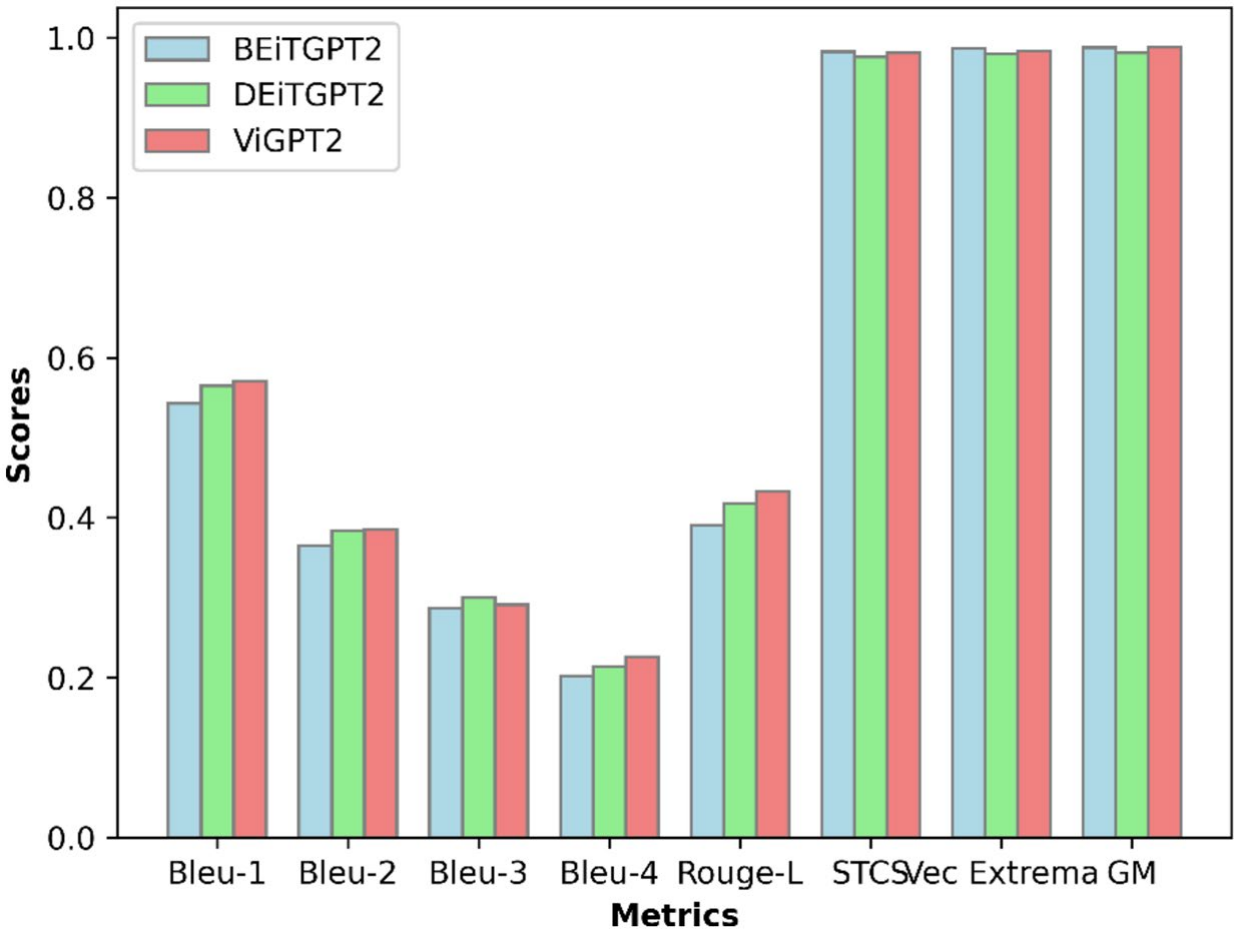
Evaluation metrics (Word overlap and semantic answer similarity metrics).

For model evaluation, we have applied Natural Language Generation evaluation metrics for the generated sequence of tokens from the images. For model evaluation word overlap metrics are used in this work. But word overlap metrics are not only sufficient to evaluate the proper performance of the model since the generated sequence consists of words that are different but the actual meaning of the generated would be similar. So, two types of evaluation metrics word overlap metrics and semantic similarity metrics are used^33^. Word overlap metrics consist of Bleu scores Eq. (9), Rouge L Eq. (10) are used. Originally developed for assessing the quality of machine translation outputs, Bleu scores have found widespread application in various text generation tasks, including automatic report generation from medical images. Semantic similarity metrics consist of skip-thought cosine similarity Eq. (11), vector extrema which is the extreme value of skip thought cs, and greedy matching. Skip Thought Cosine Similarity is a metric used to measure the similarity between two sentences based on the embeddings generated by a pre-trained language model^33^. In vector extrema, the element-wise maximum and minimum values of the embeddings for each dimension are computed from the language model, and the cosine similarity is calculated between them. Greedy matching with correlation coefficient is a technique used to measure the similarity between two sequences of tokens based on their order and correlation^15^. Table 3 shows the word overlap metrics of our model and it is compared with the existing models. Table 4 shows the semantic similarity metrics of our proposed models. It shows that our models perform better generation of reports from the existing language models. Figure 8 shows the evaluation metrics of our models.

9 $$BLEU = BP \times \text{ exp} \left(\frac{1}{N} \sum_{n=1}^{N}{\text{log}}\left({prec}_{n}\right)\right),$$

where BP is the brevity penalty, N is maxed n-grams, and price is the precision of n-grams.

10 $$Rouge{\text{-}}L = \frac{LCS \left(C, R\right) }{R},$$

where LCS (C, R) is the longest common subsequence of the candidate and reference.

11 $$Skip\, thought CS = \frac{model(s1)\cdot model(s2)}{\left|model(s1)\right|\cdot \left|model(s2)\right|},$$

where S1 and S2 are the vectors of the original and generated sentences, the model is the language model.

**Table 3:** Model performance analysis.

Model	BLEU 1	BLEU 2	BLEU 3	BLEU 4	ROUGE-L
MvH (2019)^1^	0.529	0.315	0.255	0.143	0.423
CNN-RNN (2023)^2^	0.497	0.319	0.23	0.174	0.399
Multi modal RNN (2018)^3^	0.464	0.358	0.27	0.195	0.366
Memory driven (2020)^5^	0.47	0.304	0.219	0.165	0.371
Hierarchical RNN (2019)^25^	0.436	0.278	0.197	0.15	0.341
Mul-Attr (2022)^12^	0.496	0.327	0.238	0.178	0.381
UAR (2023)^13^	0.53	0.365	0.263	0.2	0.405
TrMRG (2022)^14^	0.532	0.344	0.233	0.158	0.387
KERP (2019)^15^	0.482	0.234	0.143	0.096	0.339
Srinivasan et al. (2020)^7^	0.464	0.301	0.212	0.158	–
PoKE (2021)^8^	0.36	0.224	0.149	0.106	0.284
BEiTGPT2 (Our model)	0.543	0.365	0.287	0.202	0.391
DEiTGPT2 (Our model)	0.565	0.384	**0.3**	0.214	0.418
ViGPT2 (Our model)	**0.571**	**0.385**	0.291	**0.226**	**0.433**

Significant values are in bold.

**Table 4:** Semantic answer similarity validation.

Model	Skip thought CS	Vector extrema	Greedy matching
BEiTGPT2	0.9829	0.9871	0.9878
DEiTGPT2	0.9761	0.98	0.9813
ViTGPT2	0.9811	0.9836	0.9882

### Ablation study

**Figure 9:**
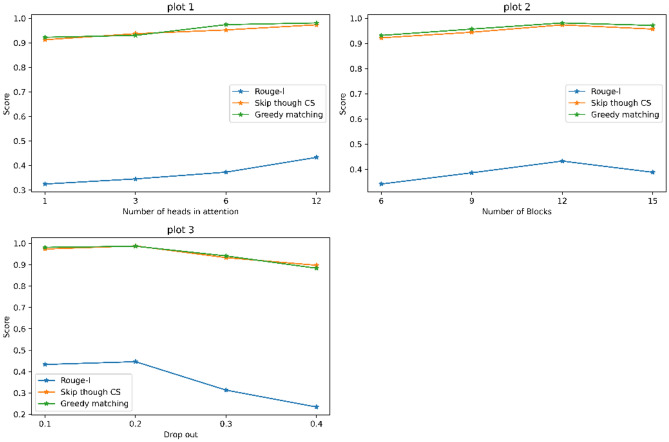
Ablation study scores for no of heads, no of blocks, and drop out in attention.

This section details an ablation study conducted to analyze the impact of various hyperparameters on the performance of the ViTGPT2 architecture for medical report generation. The study focuses on evaluating and identifying the parameters of the network that are crucial for generating medical reports. Our study focuses on four hyperparameters. Figure 9 shows the effect of the model on varying hyperparameters.

#### Number of heads in multi-head attention (MHA)

The MHA layer plays a crucial role in ViTGPT2, allowing the model to attend to relevant parts of the input sequence. This study examines the effect of varying the number of heads (1, 3, 6, 12) within the MHSA layer. Table 5 shows that increasing the number of heads might improve the model’s capability to capture intricate relationships within the medical data, but it could also lead to overfitting with too many heads.

**Table 5:** Number of heads hyperparameter.

No. of heads	Rouge-l	Skip through CS	Greedy matching
1	0.3246	0.9128	0.9227
3	0.3454	0.9373	0.9305
6	0.3729	0.9527	0.9743
12	0.4332	0.9736	0.9813

#### Number of encoder and decoder blocks

The encoder–decoder architecture is fundamental to ViTGPT2. The encoder processes the input medical record, and the decoder generates the corresponding report. This study investigates the influence of changing the number of encoder and decoder blocks (6, 9, 12, 15). Table 6 shows that a higher number of blocks might allow the model to learn more complex representations of the medical data, but it could also increase training time.

**Table 6:** Number of blocks hyperparameter.

No. of blocks	Rouge-l	Skip through CS	Greedy matching
6	0.3422	0.9222	0.9318
9	0.3867	0.9443	0.9569
12	0.4332	0.9736	0.9813
15	0.3887	0.9568	0.9715

Dropout rates: Dropout is a regularization technique used to prevent overfitting in neural networks. This study explores the impact of varying dropout rates (0.1, 0.2, 0.3, 0.4) applied to four key areas: Attention Mechanism Dropout: This controls the dropout rate for the attention weights within the MHSA layer. Residual Network Dropout: This regularizes the residual connections within the encoder and decoder blocks. MLP Dropout: This applies dropout to the MLP (multi-layer perceptron) component within each encoder and decoder block. Embeddings Dropout: This regularizes the word embeddings used by the model. Increasing the dropout value results in a drop in rouge-l and similarity scores as shown in Table 7. It is found that the dropout of 0.1 and 0.2 is optimal for VITGPT2 architecture.

**Table 7:** Dropout hyperparameter.

Drop out	Rouge-l	Skip through CS	Greedy matching
0.1	0.4332	0.9736	0.9813
0.2	0.4463	0.9881	0.9867
0.3	0.3133	0.9332	0.9413
0.4	0.2342	0.8976	0.8836

#### Patch size for the input image

ViT models typically rely on positional encoding to inject spatial information about the patches into the model. Smaller patch sizes can make positional encoding more effective, as there’s a smaller distance to encode between related patches. Patch sizes of 16 × 16 and 32 × 32 have been studied and results show that 16 × 16 is slightly more effective than the 32 × 32 patch size and it can reduce the training complexity as shown in Table 8.

**Table 8:** Patch size hyperparameter.

Patch size	Rouge-l	Skip through CS	Greedy matching
16 × 16	0.4463	0.9881	0.9942
32 × 32	0.4142	0.9645	0.9853

### Quantitative analysis

**Figure 10:**
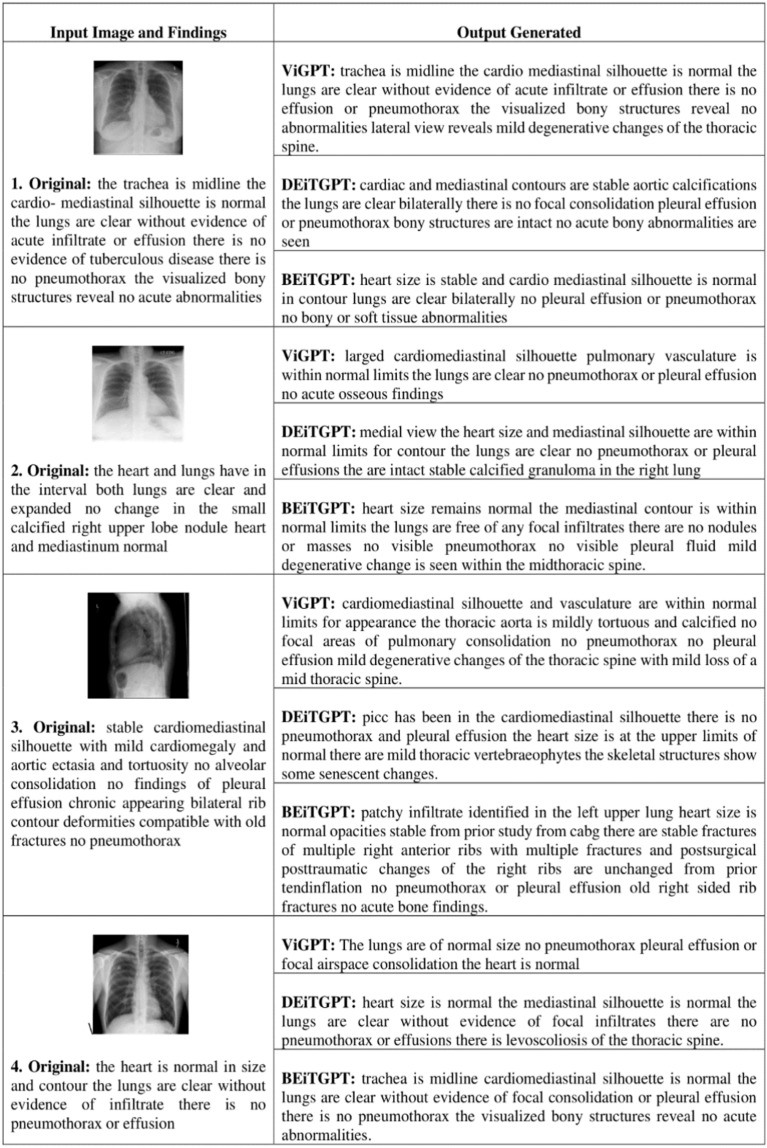
Generated output.

**Figure 11:**
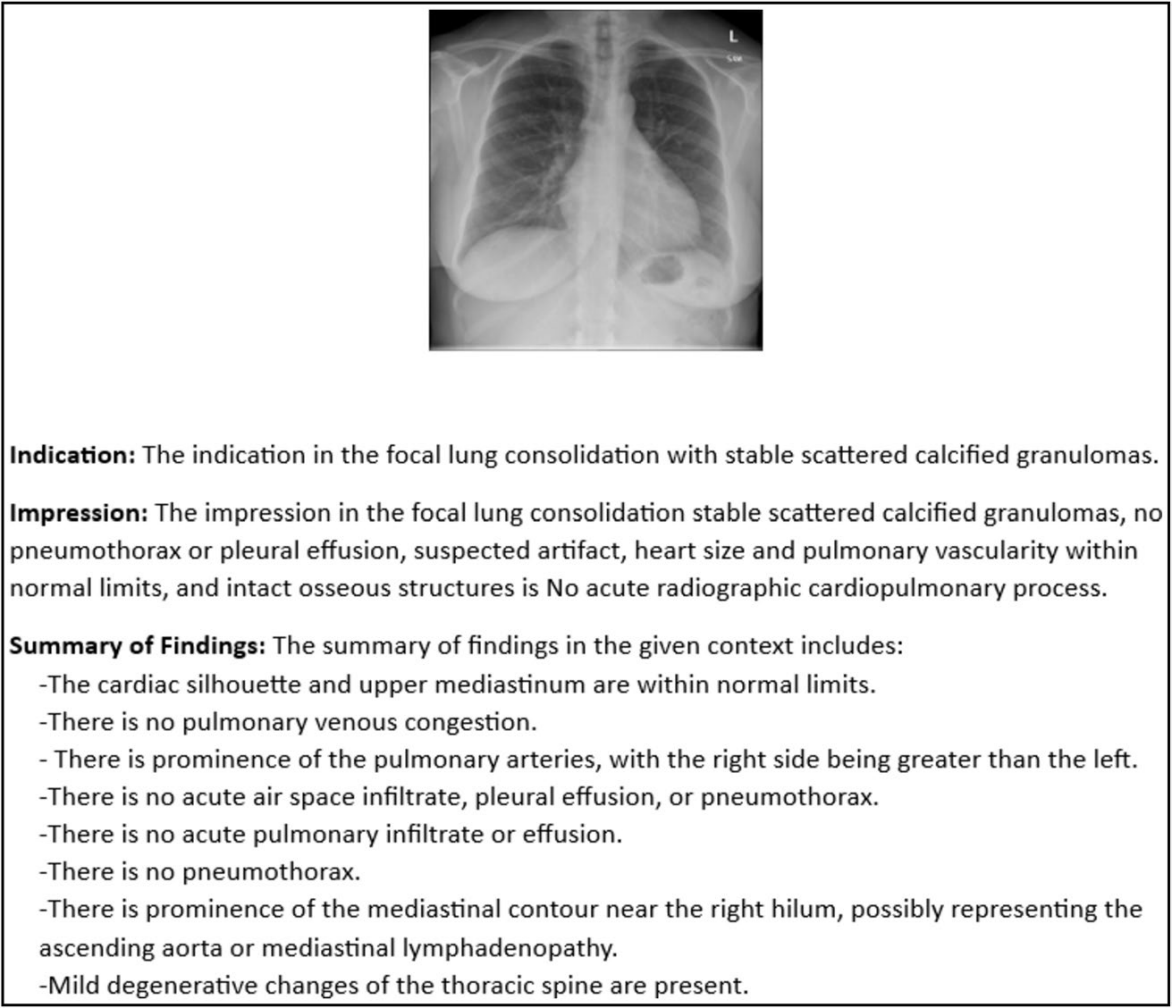
Generated report.

In this section, a few samples of medical images are tested with our model and analysis has been performed based on the Skip thought Cosine similarity, RAG answer correctness, and RAG answer similarity as shown in Table 9. Cases mentioned in Table 9 are the scores respective to the test cases of generated output from the ViTGPT2, BEiTGPT2, and DEiTGPT2 models from Fig. 10. RAG answer correctness depends on the word overlap attributes like precision, and recall of the generated words, whereas RAG answer similarity depends on the cosine similarity of the retrieved output and ground truth values. Generated outputs are passed on to the lang chain Chroma vector embeddings to generate a comprehensive report with the additional knowledge. Figure 11 shows the generated report from the lang chain module which contains indication, impression, and summary of the findings.

**Table 9:** Test cases analysis.

Test case no.	Model	Skip thought CS	RAG answer similarity	RAG answer correctness
Test case 1	ViGPT2	0.976686	0.927576	0.721483
	DEiTGPT2	0.993528	0.897711	0.621039
	BEiTGPT2	0.995373	0.875159	0.522198
Test case 2	ViGPT2	0.975778	0.893751	0.494292
	DEiTGPT2	0.985377	0.942548	0.64892
	BEiTGPT2	0.973291	0.912141	0.531985
Test case 3	ViGPT2	0.982428	0.915123	0.628129
	DEiTGPT2	0.941918	0.871831	0.533929
	BEiTGPT2	0.981342	0.921913	0.593288
Test case 4	ViGPT2	0.983156	0.912382	0.583743
	DEiTGPT2	0.991838	0.931561	0.673849
	BEiTGPT2	0.983137	0.923817	0.712237

**Figure 12:**
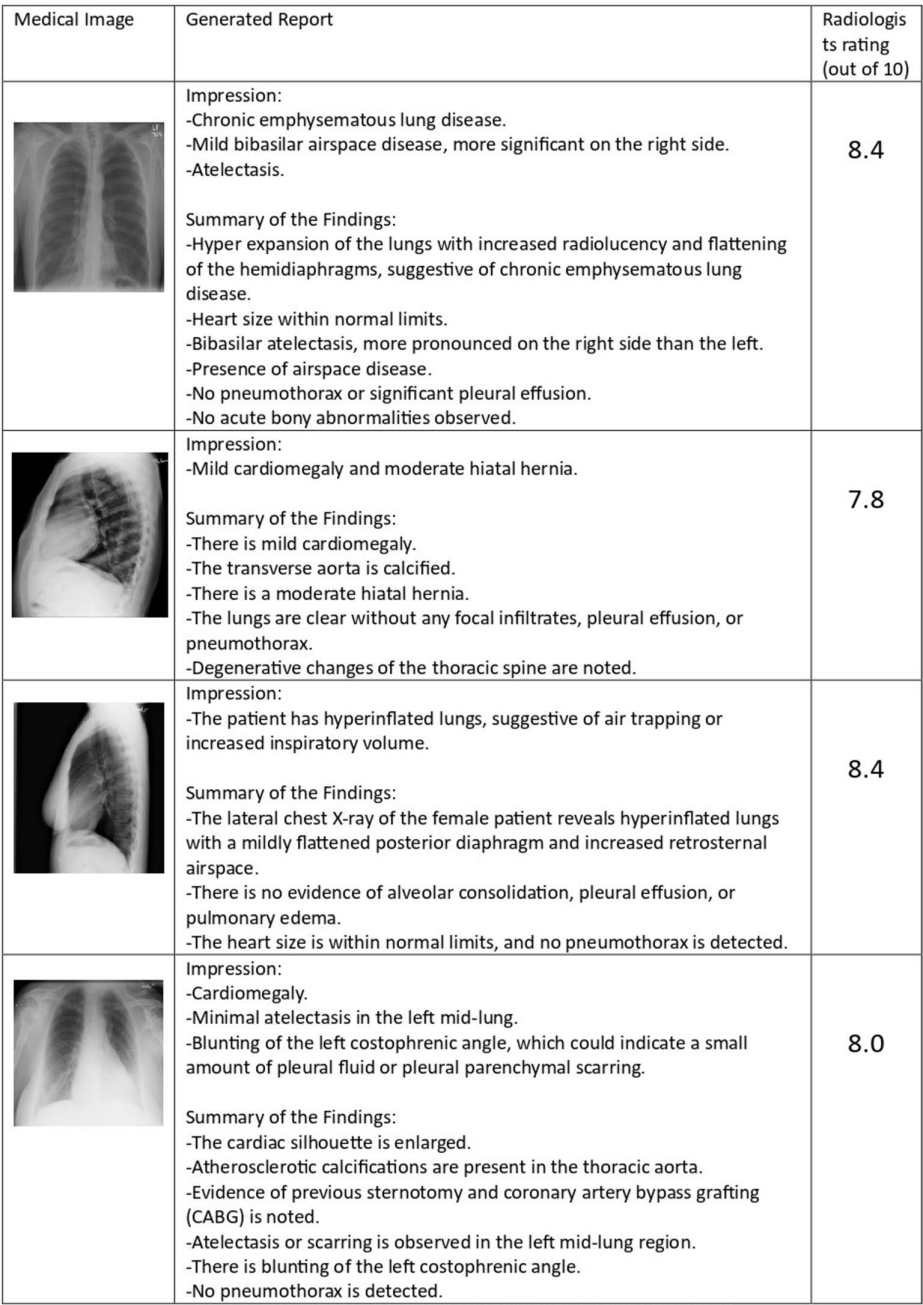
Radiologists’ correctness rating on the generated reports.

This section also provides a quantitative analysis from the radiologists and medical practitioners. A Google form with results of 10 generated reports for 10 images was circulated to medical practitioners to give the rating for the correctness of the report generated. A total of 5 responses were recorded from 3 radiologists and 2 doctors and the average report correctness rating from them and their feedback was taken as parameters to assess the quality of the generated reports. They have given feedback that these reports demonstrate high accuracy and detail, clearly outlining key findings with appropriate terminology. Reports provide thorough and concise assessments, making them highly useful for clinical purposes. Minor refinements could further improve their quality. Another examiner gave feedback that the impressions drawn from the findings were logical. While these reports are very useful in clinical settings, a few minor improvements in the explanation of findings or additional contextual details could elevate them to an exemplary level. Figure 12 gives the details of the rating given by 4 doctors.

## Limitations

The dataset used, the Indiana University X-ray dataset, contains a specific set of chest X-ray images with limited abnormalities and conditions. This narrow scope restricts the model’s ability to generalize to other types of medical images or X-rays depicting a wider range of abnormalities. The Vision Transformer (ViT), its variants (DEiT and BEiT), and GPT-2 are large models that require significant computational resources for training. Due to limited access to high-performance computing resources, such as those available through Google Colab GPU, training these models to their full potential is challenging. This constraint can lead to suboptimal model performance. Medical reports are highly dependent on the individual practitioner’s expertise, style, and interpretation of the images. This variability introduces a level of subjectivity that can affect the consistency and accuracy of the generated reports. The model may capture this subjectivity, leading to inconsistent or erroneous outputs. Generating accurate medical reports requires a deep understanding of medical terminology and the ability to contextually apply this language. While the model utilizes advanced transformer architectures, capturing the nuance and specificity of medical language remains challenging. The evaluation metrics used focus on word overlap and textual similarity. These metrics may not fully capture the clinical relevance and accuracy of the generated reports. Human evaluation by medical experts is necessary to assess the clinical utility of the reports, which is not feasible within the scope of this project. Integrating the automated report generation system into real-world clinical workflows poses significant challenges. It requires rigorous validation, user acceptance, and seamless integration with existing medical imaging systems and electronic health records.

## Future scope

The future scope of the proposed automated report generation model is vast and promising. Expanding the dataset to include a broader range of medical imaging modalities such as CT scans, MRIs, and ultrasounds, as well as a more comprehensive array of abnormalities and conditions, will enhance the model’s generalizability and applicability. Incorporating data augmentation techniques can further address data imbalance issues. Improving model architectures by exploring hybrid models that combine transformers and developing more resource-efficient variants will enhance feature extraction and make the model more accessible in low-resource settings. Additionally, integrating advanced natural language processing techniques to better handle medical terminology and incorporating human-in-the-loop systems for real-time feedback and validation can significantly improve the model’s accuracy and reliability. Expanding evaluation metrics to include clinical relevance and qualitative assessments by medical professionals will ensure the model’s practical utility in real-world clinical workflows.

## Conclusion

Our research represents significant work in the field of automatic report generation from medical images, offering a comprehensive framework that amalgamates cutting-edge techniques from image feature extraction and natural language processing domains. At the core of our contributions lies the development of a novel multi-modal transformer-based architecture, leveraging the capabilities of Vision Transformer (ViT), BEiT, and DEiT in addition to the Generative Pre-trained Transformer 2 (GPT-2). This innovative framework surpasses traditional Recurrent Neural Network (RNN) models, yielding superior performance in generating precise and contextually relevant medical reports. The integration of a cross-attention mechanism between the structural information of medical reports and image features represents a pivotal advancement in the field. Through this mechanism, our framework effectively bridges the gap between visual and textual information, enabling a good understanding of the underlying medical conditions. We have evaluated the model with semantic similarity and only a few papers have mentioned their model performance with semantic answer similarity (SAS) in their papers. We have achieved unprecedented levels of SAS scores and informativeness in the generated reports.

Our work also underscores the importance of comprehensive evaluation methodologies in assessing the efficacy of automatic report-generation systems. By employing a diverse set of evaluation metrics, including word overlap metrics and semantic metrics, we have provided quantitative insights into the performance of our models. Moreover, the integration of general knowledge into the generated reports through the Chroma vector store and Lang chain represents a significant enhancement in content richness and relevance. By augmenting the domain-specific information with broader contextual knowledge, our framework ensures that the generated reports are not only accurate but also informative and actionable for medical practitioners. Our research shows good potential in automatic report generation from medical images, offering a robust and scalable solution that holds immense potential for revolutionizing the healthcare sector.

### Supplementary Information

Supplementary Information.

## Data Availability

We are using the Open-I collection of the Indiana University X-ray dataset https://openi.nlm.nih.gov/faq#collection from the Indiana University hospital network as a base dataset for medical Imaging analysis and report generation work^26^. This dataset contains 7470 X-ray images originally in the Dicom standard form which is a representation of the digital medical images and 3851 patients reports. Every image in the dataset consists of two views frontal and lateral view. The number of X-ray images per report varies from 1 to 5.

## Code availability

The code developed is Python notebooks and is provided as Supplementary Files while submission.
